# Mobile Phone Access and Preference for Technology-Assisted Aftercare Among Low-Income Caregivers of Teens Enrolled in Outpatient Substance Use Treatment: Questionnaire Study

**DOI:** 10.2196/12407

**Published:** 2019-09-26

**Authors:** Stacy R Ryan-Pettes, Lindsay L Lange, Katherine I Magnuson

**Affiliations:** 1 Department of Psychology and Neuroscience Baylor University Waco, TX United States

**Keywords:** mobile phones, text messaging, substance use treatment, mhealth, parenting, aftercare

## Abstract

**Background:**

Improvements in parenting practices can positively mediate the outcomes of treatment for adolescent substance use disorder. Given the high rates of release among adolescents (ie, 60% within three months and 85% within one year), there is a critical need for interventions focused on helping parents achieve and maintain effective parenting practices posttreatment. Yet, research suggests that engaging parents in aftercare services is difficult, partly due to systemic-structural and personal barriers. One way to increase parent use of aftercare services may be to offer mobile health interventions, given the potential for wide availability and on-demand access. However, it remains unclear whether mobile phone–based aftercare support for caregivers of substance-using teens is feasible or desired. Therefore, formative work in this area is needed.

**Objective:**

This study aims to determine the feasibility and acceptability of mobile phone–based aftercare support in a population of caregivers with teens in treatment for substance use.

**Methods:**

Upon enrollment in a treatment program, 103 caregivers completed a mobile phone use survey, providing information about mobile phone ownership, access, and use. Caregivers also provided a response to items assessing desire for aftercare services, in general; desire for mobile phone–based aftercare services specifically; and desire for parenting specific content as part of aftercare services. Research assistants also monitored clinic calls made to caregivers’ mobile phones to provide an objective measure of the reliability of phone service.

**Results:**

Most participants were mothers (76.7%) and self-identified as Hispanic (73.8%). The average age was 42.60 (SD 9.28) years. A total of 94% of caregivers owned a mobile phone. Most had pay-as-you-go phone service (67%), and objective data suggest this did not impede accessibility. Older caregivers more frequently had a yearly mobile contract. Further, older caregivers and caregivers of adolescent girls had fewer disconnections. Bilingual caregivers used text messaging less often; however, caregivers of adolescent girls used text messaging more often. Although 72% of caregivers reported that aftercare was needed, 91% of caregivers endorsed a desire for mobile phone–based aftercare support in parenting areas that are targets of evidence-based treatments.

**Conclusions:**

The results suggest that mobile phones are feasible and desired to deliver treatments that provide support to caregivers of teens discharged from substance use treatment. Consideration should be given to the age of caregivers when designing these programs. Additional research is needed to better understand mobile phone use patterns based on a child’s gender and among bilingual caregivers.

## Introduction

Adolescent substance use is a major public health concern in the United States. The high rates of substance use coupled with significant unmet treatment needs and the alarming rates of recurrence of use are concerning. Recent estimates suggest that 2.0 million adolescents in the 12- to 17-year age group used illicit substances in 2017. Among these users, 1.0 million needed substance use treatment. Strikingly, among those who complete treatment, research suggests that 60%-70% will have a recurrence of use within 90 days after a treatment episode and 85%, within 1 year following treatment [1-3].

Improvement in parenting practices can positively mediate the outcomes of outpatient treatment for adolescent substance use disorder [4,5], and these findings extend to the aftercare period. Research suggests that evidence-based treatments for adolescent substance use are successful at improving parenting practices and that changes in monitoring and positive parenting behaviors mediate relations between treatment and adolescent substance use outcome [4,6]. There is evidence to suggest that parenting practices during the aftercare period also mediate adolescent substance use outcomes. To date, one study has examined parenting practices as a mechanism of change during aftercare [7]. Results of this study showed that the combination of continuing care and parent involvement is related to better adolescent substance use outcomes. Results of nonmediation studies of the posttreatment period suggest that these results may extend beyond parental involvement. For example, Stanger and colleagues [8] found that parental monitoring at the end of outpatient treatment was related to abstinence among adolescents after treatment. In a follow-up study of a low-income, higher-proportion, minority sample, Stanger and colleagues [8] included posttreatment booster sessions for parents, hypothesizing that aftercare for parents would be related to maintenance of effective parenting practices, resulting in higher adolescent rates of abstinence [9]. The investigators were unable to test this hypothesis due to poor parental attendance at the booster sessions. Results showed that parents attended an average of less than one session over a 3-month period and that the rates of adolescent relapse were significant. Caregiver participation in aftercare may be improved when aftercare is transitioned to the community; however, previous research on aftercare following outpatient services has not disentangled child and caregiver rates of participation [10].

Research suggests that aftercare services for parents of youth discharged from outpatient substance use treatment are important; however, offering clinic-based aftercare services may not be effective due to poor participation [9]. Parental participation in clinic-based aftercare may be poor, in part, due to significant barriers [11,12]. Common systemic-structural barriers to parent participation in mental health services include indirect financial cost (eg, lost wages for missed work) and lack of flexibility of appointments and settings (ie, clinic-based). Although moving face-to-face aftercare to the home setting may improve attendance among families [10], low-income caregivers may still find it difficult to fully engage in sessions [13,14]. Low-income caregivers may experience insurmountable barriers to clinic- and home-based services. For example, inadequate support, poor parental efficacy, low hourly wages, and significant daily hassles [15] may make it exceptionally difficult to pool resources or miss work due to the downstream effects.

Barriers to treatment participation is a major contributing factor in observed socioeconomic health disparities [16]. Research shows that while low-income adolescents are not more likely to use drugs [17], they are more likely to develop a problem and face the consequences of substance use due to differences in initiating treatment [16,18], engaging in treatment [16,18], and attending aftercare support [19]. One way to improve adolescent rates of abstinence and decrease socioeconomic health disparities in the area of adolescent substance use may be to increase the availability of aftercare services via cost-effective technologies that are far reaching, are on demand, and target effective parenting strategies.

Given that mobile phone ownership and usage are omnipresent in our society [20], one of the many advantages of mobile health (mHealth) interventions is their ability to provide widely available, far-reaching, and on-demand treatments to individuals facing significant barriers to receiving face-to-face services. Successful adoption of mobile phone–based aftercare for low-income parents is predicated upon access and reliability of mobile phone service, desire to engage in mobile phone–based aftercare support, and belief that a mobile phone–based program with parenting support is needed.

Although research has established that low-income populations have access to mobile phones [20-23] and that ownership may be significantly higher among low-income caregivers of children than national averages [24], there are at least two gaps in this research. First, our review of the literature failed to find reports of access to mobile phones among low-income caregivers of children involved in substance use treatment. Low-income caregivers of children involved in substance use treatment face more economic strain than low-income caregivers of children not involved in substance use [25]. This strain may impact access to mobile phone technology and reliability of service. Indeed, rates of mobile phone access among adult substance users is lower than the national averages [21-23]. Second, prior research has relied on participants’ self-report of reliability of service [21-23]. Research has yet to include objective indicators of reliability of service in research characterizing mobile phone ownership in low-income populations. A better understanding of access and reliability of service among low-income caregivers of adolescents involved in substance use treatment is needed to better understand the feasibility of using mHealth interventions with this population.

Although research has established that low-income caregivers of children desire mHealth interventions [26,27], this research has been limited to caregivers of children with medical conditions. Preference for mHealth interventions among caregivers of substance-using teens is not available. Successful adoption of mobile phone–based interventions with low-income caregivers of teens exiting treatment for substance use requires an understanding of whether caregivers desire to engage in mobile phone–based support. Attitudes toward mHealth interventions for medical conditions may not be shared toward mHealth interventions for substance use. Two main reasons weaken transferability of attitudes. First, interventions for medical conditions are viewed as less stigmatizing than interventions for substance use [28]. Second, caregivers may denounce the need for their own mHealth intervention because it is the child who is struggling with substance use—an attitude that is pervasive in standard substance use counseling for teens [29]. Therefore, it is critical to understand whether caregivers of substance-using teens desire mHealth interventions.

Currently, there is no research on whether caregivers of youth who use substances believe that a mobile phone–based program with parenting support would meet their needs. A critical review and empirical test of the mobile health service adoption model suggests that perceived usefulness is both directly related to behavioral intention to use mHealth services and indirectly related to behavioral intentions to use mHealth services through an individual’s feelings about performing the target behavior [30]. Given that the goal of designing mHealth services is user adoption, it is critical to understand whether caregivers would find a mobile phone–based program with parenting support useful.

To address the current gaps in the literature and offer formative work for the design of mHealth interventions for parents of youth in substance use treatment, this study recruited caregivers of low-income status who were attending behavioral family therapy for adolescent substance use to characterize mobile phone ownership and use; assess self-report and objective data of reliability of mobile phone service; examine caregiver desire for mobile phone–based aftercare support; and examine specific content caregivers desire as part of a mobile phone–based aftercare program focused on parenting skills.

## Methods

### Participants and Study Overview

Caregivers of teenagers (N=103) enrolled in an outpatient substance abuse treatment program affiliated with an academic institution in the Southwestern region of the United States participated in this study, which concluded in July 2016. For the purpose of this study, a caregiver was defined as an adult who has the legal authority to make treatment decision for the enrolled teenager, an adult who makes decisions about the enrolled teenager, or an adult who makes sure the teenager is looked after every day. Eligible caregivers were associated with teens under the age of 18 years who were actively participating in an outpatient treatment program for a substance use disorder. The survey information reported herein was collected as part of procedures for the development of an outpatient clinic devoted to delivering substance use treatments to low-income teenagers and their caregivers and for development of mobile tools capable of enhancing the effects of treatment and sustaining treatment gains. Upon admission, all families were presented with a consent to treatment form that included information about the goals of the clinic, a rationale for surveys included in their admissions packet, and potential uses of their survey and clinic data. Participants had the opportunity to allow or disallow their survey and clinic data to be used in research. All families understood that they could receive services irrespective of their decision. The local institutional review board approved this study.

### Procedure

Completion of a mobile phone ownership, usage, and preference survey via paper and pencil (n=19) or a computer (n=84) was part of admission to an adolescent outpatient substance use treatment program that provides service to families that are uninsured, receiving Medicaid benefits, or earning <US $20,000 per year. Upon discharge from treatment, research assistants completed a chart review to obtain survey results and code the outcome of outbound calls made to mobile phones by clinic staff. Data to describe the population, also collected via chart review, included demographic information and primary substance of choice for the enrolled teenager.

### Measures

#### Client Information and Substance Use History

As part of the admissions process, caregivers completed a client information form and their respective teenager completed a substance use history questionnaire. For the purpose of this study, only data necessary to describe the sample was collected during chart review. Parents provided their age, relationship to the enrolled teenager, languages spoken, and preference for spoken language in an open-text field. Parents selected their race (white, black/African American, American Indian/Alaskan Native, Native Hawaiian/other Pacific Islander, Asian, more than one race, or other), ethnicity (Hispanic or non-Hispanic), and education (less than seventh grade, junior high, partial high school, high school graduate, partial college, college degree, or graduate/professional training) from a list of options. Enrolled teenagers provided the name of their primary substance of choice in an open-text field on a substance use history questionnaire.

#### Mobile Phone Ownership, Usage, and Preference Survey

The questionnaire consisted of 18 items replicating the surveys conducted by McClure and colleagues [21] and Milward and colleagues [22], which covered availability of a mobile phone, type of phone, service plan, and day-to-day use of mobile phone features. To extend previous surveys, additional questions were developed by the first author to assess preference for aftercare support, in general, and specifically via mobile phone and preference for the content of mobile phone-based support. Surveys were available in English or Spanish, and caregivers selected the version according to their primary language preference.

#### Access and Reliability of Service

In addition to self-report of accessibility and reliability of service, an objective measure of accessibility and reliability of service during treatment was obtained. This measure included a chart review of outbound calls made to caregivers who completed the survey. These caregivers reported that they owned their mobile phone and indicated that they intended to use the mobile phone as their primary means of communication with the clinic (100%). Clinic confirmation of appointment policy included contacting caregivers 3 days before a scheduled appointment and on the day of a scheduled appointment. All outgoing phone calls were entered into a telephone contact and appointment log specifying the time, date, phone number used to make contact, and outcome by clinic staff. Clinic staff recorded the outcome of outgoing calls as either “confirmed,” “left a message,” “unable to leave a message, yet ringing,” or “phone disconnected.” Second author LL completed all chart reviews and created a data file for review by the first author SR-P. All data were checked and verified as correct by the first author.

Accessibility was operationalized as the percent of contact calls determined to be in-service (ie, outbound calls recorded as confirmed, left a message, or unable to leave a message yet ringing). Reliability of service was operationalized as the number of days between disconnection and when the mobile phone was able to accept clinic calls, and treatment staff was able to either leave a message or speak with the individual. Through this method, researchers were able to calculate the number of days until the clinic was able to either leave a message or talk with the client, after an unsuccessful attempt due to disconnection. Three call outcomes were missing and left as missing data.

### Statistical Analyses

Data were analyzed using SPSS (Version 23. IBM Corp, Armonk, NY). Descriptive statistics were used to quantify demographic information and mobile phone characteristics, utilization, and accessibility and reliability of service. Consistent with previous research [21,23], exploratory binary logistic regression and linear regression analyses were conducted to examine the association between demographic variables and mobile phone characteristics, utilization, and accessibility and reliability of service.

For binary logistic regression analyses, select mobile phone characteristics, use preferences, and self-report of accessibility and reliability of service were regressed on parent age (continuous variable), ethnicity (non-Hispanic=0, Hispanic=1), bilingual language (nonbilingual=0, bilingual=1), and education (less than high school=0, more than high school=1) as well as child age (continuous variable) and gender (female=0, male=1) in separate regression models. The mobile phone characteristics and use preference variables were selected a priori as those that would provide developers information about accessibility and reliability of service as well as mobile features to consider incorporating when tailoring mHealth services: yearly contract (no=0), primarily use phone for text messaging (no=0), phone number changed one or more times in the past year (no=0), accesses to the internet mostly from the mobile phone (no=0), and experience with service connection issues (never or rarely=0). For linear regression analyses, continuous variables indicating inaccessibility and disruption of service (ie, number of times unable to leave message and number of times disconnected) were regressed on the same demographic variables as those used in the logistic regression analyses.

An alpha of .05 was maintained throughout. Considering these parameters, power analysis was conducted using the online estimation tool GPOWER (Version 31. Heinrich-Heine-Universität Düsseldorf, Düsseldorf, Germany) [31]. Results showed that our sample size was adequate to detect results of the medium effect size for linear regression analyses.

## Results

### Participants

Demographic information for caregiver and teen participants is presented in Table 1. Caregivers had a mean age of 42.60 (SD 9.28) years. The majority of caregivers were mothers (76.7%; biological, step, or adoptive), identified as Hispanic (73.8%), and reported English as their primary spoken language (88.3%). Teens participating in treatment had an average age of 15.94 (SD 1.32) years. Most teens were male (65%), enrolled in high school (71.8%), and primarily used marijuana (94.2%).

**Table 1:** Caregiver and child demographics (N=103).

Variable			Value
**Caregiver variables**			
	Age of parent, mean (SD)		42.60 (9.28)
	**Relationship to client, n (%)**		
		Biological/step/adoptive mother	79 (76.7)
		Biological/step/adoptive father	14 (13.6)
		Grandmother	5 (4.9)
		Other (aunt, adult sibling)	5 (4.9)
	**Race, n (%)**		
		White	73 (70.9)
		Black/African American	9 (8.7)
		American Indian/Alaskan Native	1 (1.0)
		Native Hawaiian/Other Pacific Islander	0 (0.0)
		Asian	0 (0.0)
		More than one race	6 (5.8)
		Other, not specified	12 (11.7)
	**Ethnicity, n (%)**		
		Hispanic	76 (73.8)
	**Primary language, n (%)**		
		English	91 (88.3)
		Spanish	12 (11.7)
		Bilingual (English/Spanish)	49 (47.6)
	**Education^a^, n (%)**		
		Less than seventh grade	2 (1.9)
		Junior high	9 (8.9)
		Partial high school	14 (13.9)
		High school graduate	34 (33.7)
		Partial college	29 (28.7)
		4-year college degree	10 (9.9)
		Graduate/professional training	3 (3.0)
**Child variables**			
	Age of child, mean (SD)		15.94 (1.32)
	**Gender, n (%)**		
		Male	67 (65.0)
		Female	36 (35.0)
	**Grade level, n (%)**		
		High school	74 (71.8)
		Junior high	21 (20.4)
		Graduated	2 (1.9)
		Not in school	6 (5.8)
	**Primary substance, n (%)**		
		Marijuana	97 (94.2)
		Synthetic marijuana	4 (3.9)
		Alcohol	1 (1.0)
		Other	1 (1.0)

^a^N=101.

### Mobile Phone Characteristics and Use

Full results for mobile phone ownership, characteristics, and use are presented in Table 2. Notably, the majority of caregivers used mobile phones at least once per week (92%), reported mobile phone ownership (94.2%) of smartphones (83.5%), had unlimited text messaging (92%), and reported text messaging as the most used mobile phone feature (59%).

### Self-Report of Accessibility and Reliability of Mobile Phone Service

Full results for self-report of mobile phone accessibility and reliability of service are presented in Table 2. Notably, most caregivers maintained the same mobile phone number over the past year (64%) with infrequent connection disruptions (48% reported rare disruption and 28% reported no disruption). Additionally, positive outcomes of outbound calls from clinic staff to caregivers’ mobile phones were high, with 97.2% of calls reaching a phone that was in service and able to receive calls or text messages, including 47.2% of clients who were reached, 45.5% of clients who received a voice mail, and 4.5% who showed a missed call from the clinic (ie, phone ringing but unable to leave a message because the client’s mailbox was either full or no message had been setup), while only 2.7% of calls reached disconnected phones.

**Table 2:** Mobile phone accessibility, reliability, and use.

Caregiver variable					Value
**Accessibility, n (%)**					
	**Access (whole sample, n=103)**				
		Owns a mobile phone			97 (94.2)
		Daily access, but do not own			3 (2.9)
		Unreliable access, do not own			3 (2.9)
	**Mobile phone owners (n=97)**				
		Cell is primary phone			97 (100.0)
		Smartphone device			81 (83.5)
			**Smartphone type**		
				iPhone	13 (16.0)
				Android	57 (70.4)
				Windows	4 (4.9)
				Other	7 (8.6)
	**Service type (n=97)**				
		Pay-as-you-go			65 (67.0)
		Yearly			32 (33.0)
**Reliability (those with access, n=100), n (%)**					
	**Mobile number changed last year**				
		Never			64 (64.0)
		Once			21 (21.0)
		Twice			8 (8.0)
		More than thrice			7 (7.0)
	**Disruptions in mobile phone connections**				
		Never			28 (28.0)
		Rarely			48 (48.0)
		Sometimes			20 (20.0)
		Often			2 (2.0)
		Always			2 (2.0)
**Use (those with access, n=100)**					
	**Text message limit, n (%)**				
		No			92 (92.0)
		Yes			3 (3.0)
		Not sure			5 (5.0)
	Use mobile to text, n (%)				97 (97.0)
	Use mobile to email, n (%)				76 (76.0)
	Use mobile to take pictures, n (%)				93 (93.0)
	Use mobile to play music, n (%)				82 (82.0)
	Use mobile to download mobile applications, n (%)				84 (84.0)
	Use mobile to access internet, n (%)				92 (92.0)
	**Use mobile most for, n (%)**				
		Text			59 (59.0)
		Email			8 (8.0)
		Pictures			3 (3.0)
		Music			7 (7.0)
		Apps			4 (4.0)
		Internet			19 (19.0)
	Regular internet use (at least once a week), n (%)				92 (92.0)
	**Access internet from which device most?, n (%)**				
		Cell			58 (58.0)
		Other device			13 (13.0)
		Both equally			21 (21.0)
		Not sure			8 (8.0)
	**Outcome of outgoing clinic calls to caregivers' mobile phone (n=2776), n (%)**				
		Connected			2698 (97.2)
		Caregiver reached			1311 (47.2)
		Left message			1263 (45.5)
		Unable to leave voice message			124 (4.5)
		Disconnected			75 (2.7)
		Unknown/missing details			3 (0.1)
	**Number of unreachable days, median (range)**				
		Number of disconnected days			14 (2)
		Number of unable to leave voice message days			28 (2)

### Preference for Mobile Phone–Based Services and Support

When queried, only 72% of caregivers endorsed the need for nonspecific aftercare support; however, 91% of caregivers endorsed the desire for text messaging–based aftercare support (Table 3). Caregivers reported that text messages with the following content would be helpful: ways for improving communication with their child (63%), reminders and encouragement to use consequences (62%), suggestions for getting their teen involved in positive activities (62%), and messages with tips for monitoring their teen’s substance use (56%). Caregivers also reported the desire for additional counseling for the child (32%) and general family/caregiver support (26%). Overall, 70.3% of caregivers preferred receiving texts 1-3 times weekly, 22% preferred daily, and 7.7% preferred 4-5 times weekly.

### Demographic and Mobile Phone Relationships

Regression results showing relationships of caregiver and teen demographics with mobile phone characteristics, accessibility, and use patterns are presented in Tables 4 and 5. Younger caregivers were significantly more likely to have pay-as-you-go mobile phone contracts (β=0.06, *P*=.03), have a higher number of phone disconnections during treatment (β=–0.03, *P*=.04), and use their phone to access the internet (β=–0.07, *P*=.009). In addition, bilingual caregivers were significantly less likely to use texting as their main mobile phone feature (β=–0.87, *P*=.04). Caregivers with male teens were significantly more likely to have fewer disconnections during treatment (β=–0.46, *P*=.04) and those with adolescent girls were more likely to use texting as their main mobile phone feature (β=–0.99, *P*=.03).

**Table 3:** Aftercare support and clinic calls made to mobile phones (caregiver variables).

Variable			Value, n (%)
**Additional support needed (those with access, n=100)**			
	Yes	72 (72.0)	
	No	6 (6.0)	
	Do not know	22 (22.0)	
**Interested in receiving text message parenting support (those with access, n=100)**			
	Yes	91 (91.0)	
**Support focused on (those with access, n=100)**			
	Monitoring substance use	56 (56.0)	
	Using consequences	62 (62.2)	
	Positive activities	62 (62.0)	
	Communication	63 (63.0)	
**Requested text frequency (those interested, n=91)**			
	1-3 times weekly	64 (70.3)	
	4-5 times weekly	7 (7.7)	
	Daily	20 (22.0)	

**Table 4:** Logistic regressions for relations between demographics and self-report of mobile phone use, characteristics, accessibility, and reliability of service. The first group of predictors listed served as the comparison group for the binary logistic regression analyses and were coded as 0, while the second group was coded as 1. All technology outcomes were coded dichotomously with no=0 and yes=1.

Variable		Yearly contract							Text messaging used most						Phone number change once or more than once						Internet on phone							Self-report of connection issues				
		β		SE		95% CI		β		SE		95% CI		β		SE		95% CI		β		SE		95% CI		β			SE		95% CI	
**Caregiver**																																
	Age		0.06^a^		0.03		1.01-1.12		0.04		0.03		0.99-1.09		–0.04		0.03		0.92- 1.02		–0.07^b^		0.03		0.88-0.98		–0.06			0.03		0.88- 1.01
	Non-Hispanic vs Hispanic		–0.27		0.49		0.29-2.01		–0.30		0.48		0.29-1.89		1.04		0.55		0.96-8.32		–0.08		0.57		0.30-2.84		0.39			0.56		0.49-4.48
	Nonbilingual vs bilingual		–0.28		0.43		0.32-1.77		–0.87^a^		0.42		0.19- 0.95		0.30		0.42		0.60-3.06		–0.22		0.49		0.31-2.10		–0.24			0.47		0.32-2.00
	Less than versus more than high school education		0.82		0.44		0.95-5.41		–0.04		0.05		0.88-1.06		–0.01		0.01		0.97-1.02		–0.30		0.50		0.28-2.00		–0.01			0.01		0.96-1.02
**Child**																																
	Age		0.10		0.17		0.80-1.53		0.09		0.16		0.81-1.49		0.23		0.17		0.91-1.74		–0.18		0.19		0.57-1.22		0.09			0.18		0.77-1.55
	Female vs male		–0.42		0.44		0.28-1.57		–0.99^a^		0.45		0.15- 0.89		0.06		0.43		0.46-2.48		0.06		0.51		0.39-2.86		0.08			0.48		0.42-2.78

^a^*P*<.05

^b^*P*<.01.

**Table 5:** Linear regressions for relations between teen demographics and objective measures of reliability of mobile phone service. The first group of predictors listed served as the comparison group for the binary logistic regression analyses and were coded as 0, while the second group was coded as 1.

Variable		Number of times ULM^a^						Number of times disconnected					
		β		SE		95% CI		β		SE		95% CI	
**Caregiver**													
	Age		–0.01		0.01		–0.04 to 0.01		–0.03^b^		0.01		–0.05 to 0.00
	Non-Hispanic vs Hispanic		0.29		0.27		–0.24 to 0.82		0.26		0.25		–0.24 to 0.76
	Nonbilingual vs bilingual		0.00		0.23		–0.46 to 0.46		0.08		0.22		–0.35 to 0.52
	Less than versus more than high school education		0.01		0.01		–0.01 to 0.02		0.00		0.01		–0.01 to 0.02
**Child**													
	Age		–0.02		0.09		–0.20 to 0.15		–0.05		0.08		–0.22 to –0.11
	Female vs male		0.02		0.24		–0.46 to 0.49		–0.46^b^		0.22		–0.90 to –0.02

^a^ULM: unable to leave voice message.

^b^*P*<.05.

## Discussion

### Overview

Adolescent rates of return to substance use following outpatient treatment are staggering [1-3]. Research suggests that participation in aftercare and effective parenting practices posttreatment positively mediates adolescent substance use outcomes [7]; however, parent participation in aftercare is poor [9], likely due to structural and personal barriers [11,12]. One way to overcome barriers to participation is to provide parents with mobile phone–based aftercare. mHealth services are cost-effective, far-reaching, and on-demand. Further, mHealth services for low-income parents could help address socioeconomic disparities in access to aftercare services [19]. Prior to designing mHealth aftercare interventions for low-income caregivers of teens exiting treatment for substance use, it is imperative to conduct formative work to address the gaps in current knowledge. This study is the first to report mobile phone ownership, use, and accessibility and reliability of service in a low-income sample of caregivers of teens exiting treatment for substance use. Further, this is the first study to report on whether low-income caregivers of teens involved in substance use desire mHealth aftercare services. Lastly, this study provides the first report of the parenting skills caregivers would perceive as helpful if included in an mHealth aftercare program.

### Summary of Principle Results

Results of this study replicate and extend existing research. Consistent with the extant literature, our results indicate that a high percentage of low-income caregivers own mobile phones and are familiar with mobile communication technology. Extending this literature, results also demonstrate good subjective report and objective analysis of accessibility and reliability of service despite most of the sample having pay-as-you-go service. Interestingly, older caregivers were more likely to have yearly contracts and fewer mobile disconnections. Additionally, caregivers with girls in treatment used their mobile phones more often for texting and those with boys in treatment had fewer mobile service disconnections. Further extending the current literature, most caregivers reported that they would like to receive mobile phone–based support in the form of text messages following their teen’s treatment for substance use and reported interest in receiving support in areas of parenting that are common targets in evidence-based treatments.

### Technology Characteristics and Use

The rates of mobile phone ownership among caregivers in this study are comparable to the current rates in the general population [20], yet the rates were higher than those of previous reports on low-income adult populations participating in substance use treatment [21-23]. This finding suggests that among caregivers with teens participating in treatment, access to mobile phones better matches national rates. One possible explanation for these different results is that caregivers may be more likely to own mobile phones to ensure communication with their teenager and to have a broader window of availability for other authority figures that may need to reach them (eg, schools, doctors, and juvenile probation).

Similarly, the rate of smartphone ownership was higher (83.5%) in this sample than in previous reports of adults involved in substance use treatment (67% [23] and 57% [22]). Rates of smartphone ownership were also higher than the national averages (77% [20]). Two factors may account for these differences: Smartphone ownership was less common in 2014 [32], and smartphone ownership among caregivers may be higher than that of noncaregivers. High rates of smartphone ownership among caregivers may be due to greater exposure to this technology through their teens [33]. In addition, research suggests that smartphone ownership has taken the place of computer ownership [33,34]. Thus, smartphone ownership among caregivers with teens offers a way of monitoring teen activity online. In support of this explanation, overall use of smartphone technology was higher in this population than in previous studies. Specifically, 76% reported using their phone for email compared to 45% in earlier research [21]. Furthermore, 92% reported accessing the internet compared to 44% [21] and 61% [23] reported by others.

Given that more Americans own smartphones than when previous research was conducted, a more equitable comparison of technology use to other studies may be the reported use of text messaging. Nonetheless, a notable difference in the use of this communication technology was evident in this study. McClure and colleagues [21] reported that 79% of their sample had access to and used text messaging. Dahne and colleagues [23] found a discrepancy between the percentage of people who had access to text messaging (96%) and the percentage of those who actually used the text messaging feature (83%) [23]. Compared to the values reported by Dahne and colleagues [23], Milward and colleagues [22] reported a much lower percentage of adults involved in substance use treatment who used text regularly (55%). We found that 97% of caregivers used text messaging regularly and only 3% reported texting limits. These previous findings suggest that for some populations, barriers may exist to implement text message–based interventions; however, our results suggest that text message–based interventions and support may be ideal for caregivers of teenagers.

### Accessibility and Reliability of Service

Over half of the sample (65.6%) operated without a yearly mobile service contract. This result is similar to existing research of low-income adults involved in substance use treatment [22,23]. These results suggest that adults who are vulnerable to unmet treatment needs may have demographic characteristics in common, which may limit their use of yearly contracts. Similar to other studies, the majority of our participants were economically disadvantaged. However, our results for the number of times phone numbers were changed in the past year diverged from other reports—a variable considered to be a proxy measure of vulnerability for disruption in service and unreliable access to mobile phone technology. Caregivers in our study reported fewer instances of phone number changes in the past year (64% reported never) compared to other studies [37% [21] and 54% [23] reported never). This difference may also be related to their caregiver status and suggests that caregivers may be more reachable than other populations of adults involved in substance use treatment.

A unique feature of our study was the use of outbound calls made by clinic staff while families were enrolled in treatment, providing an objective indicator of disruption in mobile phone service. Results were consistent with self-report of low interruption of mobile phone service and show that participants were highly reachable despite the majority having pay-as-you-go mobile phone service. One interpretation of these results is that caregivers were reachable because of their involvement in treatment, that is, results may have shown a high rate of accessibility and service connection because caregivers were anticipating clinic calls related to appointments. Although this might have influenced outcomes, the finding that age of a caregiver is associated with reachability suggests that this explanation does not fully account for the current findings. Younger caregivers were more likely to experience disruptions in service and to have pay-as-you-go phone service. Thus, our results suggest that younger caregivers using a mobile phone–based intervention for teens involved in substance use treatment may benefit from assistance in maintaining mobile phone service. For example, mobile phone–based programs implemented by provider organizations could pay the cost of mobile service for some caregivers. The cost of maintaining mobile service during the high-risk period for adolescent relapse (first year posttreatment) costs less than continued in-person support by a mental health counselor. Overall, our results suggest that low-income caregivers of teens involved in substance use are reachable and have consistent access to the mobile phones that they own.

### Aftercare Requests

Another unique feature of our study was the inclusion of a measure of caregiver interest in aftercare services. Interestingly, only 72% of caregivers expressed an interest in aftercare support. When asked specifically about aftercare delivered via text messaging, this rate increased to 91%. Few caregivers were able to provide details of what they were interested in receiving as part of aftercare support; however, when parenting skills as a topic were specifically surveyed, more than half of the caregivers endorsed content areas consistent with evidence-based treatments for adolescent substance use, including monitoring, use of consequences, ways to initiate positive activities, strategies for communicating with teen, and encouragement and support. These results suggest a mobile phone–based program for caregivers that includes skills covered in evidence-based curricula may be well received.

### Notable Concerns

The prospect of using mobile phones to implement treatment is exciting because mobile phones offer the possibility of reducing health disparities along socioeconomic lines. Although these data suggest that low-income caregivers have access to mobile phones with reliable service, results revealed that demographic factors were related to technology use and reliability of service. First, bilingual caregivers were significantly less likely to use text messaging. This finding suggests that the specific communication technology selected for bilingual caregivers should be considered closely. Speaking two languages was unrelated to using a mobile phone to access the internet. It may be the case that digital interventions for bilingual caregivers are more likely to be adopted if delivered online. To help guide the development of programs wishing to implement a text message–based system for bilingual caregivers, additional research is necessary to better understand the reasons for lower rates of text messaging. For example, a significant proportion of this sample was bilingual but preferred English as their spoken language. A follow-up study could explore whether this finding holds true with a less acculturated sample.

Second, caregiver age may play a role in accessibility and reliability of service. Younger caregivers were significantly less likely to have a yearly contract. As noted above, it may be necessary for providers to offer social service support to caregivers based on age. Additional research is needed to determine the approximate age when caregivers are more likely to have a pay-as-you-go service plan to better target efforts in order to help caregivers maintain service during the implementation of mobile phone–based interventions. Our results also showed that caregiver age was related to the mobile communication technology most frequently used. Older caregivers were least likely to use their mobile phone to access the internet, but age was unrelated to the use of text messaging. Additional research is needed to determine treatment delivery preference. Internet-based interventions optimized for access via mobile phones may be more successful with older caregivers. Text messaging and internet-based interventions may also be equally acceptable for older caregivers.

Third, caregivers parenting teen boys involved in substance use treatment were less likely to have phone disconnections and use text messaging most often among their mobile phone technologies. Future research is needed to better understand these associations. The association between child gender and caregiver use of mobile phone technology is critical for the design of mobile phone–based interventions for caregivers of teens involved in substance use treatment. These results may be an indication of caregiver efforts to monitor and supervise their teenager, suggesting that the use of mobile technology may differ by gender. These results may also be an indication of differences in what is required to monitor teen girls versus teen boys in this age of mobile technology. Researchers and providers may need to use more engagement efforts with caregivers of teen boys involved in substance use treatment when an intervention is designed for mobile delivery.

### Limitations

Several limitations of this study should be noted, as they may impact interpretation and generalizability of the findings. First, since the goal of this study was to inform mHealth development of an aftercare service, the objective assessment of access and reliability of service may have provided different results if these data were collected once caregivers and teens were discharged from treatment. However, in a population of adults engaged in substance use treatment, Dahne and colleagues [25] showed rates of mobile phone ownership and use prior to treatment and expected ownership and use following treatment were similar among low-income adults. Additionally, in a study using ecological momentary assessment to examine medication adherence for diabetes among adolescents, researchers contacted teens through a phone call for a 10-day period following initial treatment [35]. During this time, researchers did not observe a decline in the response rate for these teens [35]. Although this period did not provide an extended assessment of reliability of mobile phone access among teens, it conveys a possible trend of consistent mobile phone access from treatment into aftercare. Importantly, children under the age of 18 years are more likely to have a mobile phone that is covered as part of a single-family plan [36], suggesting that caregivers of the youth in that study may have also experienced consistent and reliable mobile phone access from treatment into aftercare.

Second, our sample of predominately Hispanic caregivers was both a unique aspect of the study and a limitation. Minority caregivers are seldom represented in research, and there is no formative work with Hispanic caregivers of teens involved in substance use treatment. Yet, the generalization of the study results is limited. Result suggests that most caregivers in this sample were acculturated. Further, we were unable to explore associations among variables in the subsample of caregivers who identified as Hispanic.

Third, additional variables important in formative work for designing mHealth interventions for caregivers were not explored. For example, parent skills training usually includes a discussion of problematic child behaviors. We were unable to collect information about whether caregivers would want teens’ health information included in an mHealth aftercare support program.

Finally, our analyses of the association between demographic variables and mobile phone characteristics and use, accessibility, and reliability of service should be interpreted with caution, given the exploratory nature of these analyses.

### Conclusions

Results of this study suggest that the development of mobile phone–based interventions for caregivers of teens in substance use treatment is promising. The results of the survey demonstrate that mobile phone–based interventions designed for delivery using smartphone technology are feasible. Although caregivers have experience using most of the technology on their phones, usage differs by age and language. Self-report and objective data suggest that caregivers who have reliable access to mobile phones are interested in treatment delivered via mobile phones. Further research is needed to better understand the delivery of these services based on caregiver language, age, and gender of the child, as results suggest that tailoring may be needed.

## Abbreviations

mHealth: mobile health
ULM: unable to leave voice message
